# A Novel Active Imaging Model to Design Visual Systems: A Case of Inspection System for Specular Surfaces

**DOI:** 10.3390/s17071466

**Published:** 2017-06-22

**Authors:** Jorge Azorin-Lopez, Andres Fuster-Guillo, Marcelo Saval-Calvo, Higinio Mora-Mora, Juan Manuel Garcia-Chamizo

**Affiliations:** Department of Computer Technology, University of Alicante, Carretera San Vicente s/n, San Vicente del Raspeig, Alicante 03690, Spain; fuster@dtic.ua.es (A.F.-G.); msaval@dtic.ua.es (M.S.-C.); hmora@ua.es (H.M.-M.); juanma@dtic.ua.es (J.M.G.-C.)

**Keywords:** visual inspection, specular surfaces, structured lighting, quality control

## Abstract

The use of visual information is a very well known input from different kinds of sensors. However, most of the perception problems are individually modeled and tackled. It is necessary to provide a general imaging model that allows us to parametrize different input systems as well as their problems and possible solutions. In this paper, we present an active vision model considering the imaging system as a whole (including camera, lighting system, object to be perceived) in order to propose solutions to automated visual systems that present problems that we perceive. As a concrete case study, we instantiate the model in a real application and still challenging problem: automated visual inspection. It is one of the most used quality control systems to detect defects on manufactured objects. However, it presents problems for specular products. We model these perception problems taking into account environmental conditions and camera parameters that allow a system to properly perceive the specific object characteristics to determine defects on surfaces. The validation of the model has been carried out using simulations providing an efficient way to perform a large set of tests (different environment conditions and camera parameters) as a previous step of experimentation in real manufacturing environments, which more complex in terms of instrumentation and more expensive. Results prove the success of the model application adjusting scale, viewpoint and lighting conditions to detect structural and color defects on specular surfaces.

## 1. Introduction

Scientific models and theories aimed at explaining the behaviour of specular reflections are sufficiently consolidated. However, the automatic processing, using artificial vision techniques, of scenes where there are specular surfaces, has problems that have not been solved yet. Vision systems designed to deal with specular objects must cope with the optical difficulties associated with this type of material [1]. The surfaces have a high reflection coefficient which causes undesired reflections and shine, concealing, in some cases, chromatic, morphological and topographical information about the object.

Most artificial vision techniques ignore specular reflections and focus on the diffuse component of the interaction of light with objects. Thus, these techniques, which are conceived to deal with Lambertian surfaces, produce wrong results when other surfaces are in the visual scene [2,3]. The solutions put forward in the literature adopt opposite ways of approaching the problem: developing methods to detect specularities in images, in order to take advantage of existing artificial vision techniques; and designing new vision techniques that explicitly deal with these surfaces.

The methods designed for detecting specularities in the scene differ depending on the vision level in which they are approached. They take advantage of certain phenomena and characteristics of light to separate the contribution of diffuse and specular reflection: spectral distribution of reflections [4,5,6,7], polarization [8,9,10,11], analysis of the behaviour of specularities in several images [12,13,14,15,16], and combinations of them [17,18,19]. They are used either to avoid or to remove specularities in the original image. The solutions in the last case are at the low level of vision systems because reflections and shine are considered as noise to be removed. They offer a filtered image to be processed at higher levels.

Designing new vision techniques allows specular reflections to be considered as a peculiar characteristic of the surface, which allows better performance of the vision process [20,21,22,23,24,25,26]. They use mechanisms of active vision that, in some cases, are modifications or refinements of classic techniques. They are generally focused on the extraction of the object shape [27,28,29,30,31,32,33,34,35,36,37,38].

The techniques developed to deal with specular surfaces, either for detecting specularities or for extracting the object shape, must fulfill certain requirements, making them only viable in specific applications: for example, requirements related to specific electromagnetic characteristics of objects (i.e., specific methods for metallic or dielectric objects), having previous knowledge of the geometry of the scene or of the surface reflectance, etc.

Specifically, automated visual inspection is the most used tool for testing products in industry due to their ease of use and its cost [39,40,41,42]. However, there are very few vision systems that deal with the surface inspection of specular products. They share the same aforementioned drawback of the generic vision system for specular objects: they are very specific as they assume specific constraints. The lighting of the scene and the acquisition equipment are determinant factors in the proposed solutions since they help the detection of defects in images [43,44,45,46,47].

Techniques that use structured lighting appear in few systems cited in the literature. They are considered to be the most reliable and suitable for inspecting the 3D shape of products [36,48,49,50]. Moreover, they have advantage over other techniques including laser, time of flight or LIDAR because the same sensor is used to determine colour information instead of acquiring colour and shape information in an independent manner. Techniques differ in the way they acquire the lighting pattern by means of projecting on the surface [51,52,53] or focusing the system on the reflection [36,48,54,55] (assuming the object as part of optical system where lighting patterns are projected); the equipment used to generate patterns [56,57,58] (screens, projectors, etc.); and, in the method used to codify patterns [59,60,61,62,63].

Proposed solutions try to satisfy specific requirements by means of adapting to the application domain and to specific constraints of the products. A system designed to satisfy the quality control of a product generally cannot be applied to another system. As a consequence, the contribution of this paper is to provide an active vision model able to explain the problem of inspecting specular surfaces and able to help in designing vision systems for this purpose. Moreover, the paper proposes a method based on the model that is able to minimize the negative effects of specular surfaces in visual inspection and able to take advantage of specular reflections as a peculiar characteristic. The method is focused on controlling the acquisition conditions (e.g., lighting angles, viewpoints, chromaticity and other lighting characteristics, etc.) to maximize the likelihood of detecting defects. Particular characteristics of the inspection problem make control of the acquisition conditions possible.

In order to validate the model the use of simulation is proposed as an inspection system design methodology that could be systematically applied, studying conditions in which the inspection has to be carried out and designing solutions in a flexible way. Virtual inspection makes use of the virtual manufacturing technology to model and simulate the inspection process, and the physical and mechanical properties of the inspection equipment, to provide an environment for studying the inspection methodologies [64]. Simulation based on virtual imaging enables the rendering of realistic images, providing a very efficient way to perform tests compared to the numerous attempts of manual experiments [65]. The introduction of simulation provides a flexible and low-cost method (compared with experimentation in the laboratory) of testing original hypotheses and the benefits that can be drawn from this research.

The rest of this paper is organized as follows. Section 2 describes the active vision model. The method based on the model for inspecting specular surfaces is developed in Section 3. Section 5 and Section 6 evaluate the proposed method by controlling image acquisition conditions. Finally, Section 7 concludes the paper.

## 2. Active Vision Model to Deal with Specular Surfaces

We are interested in modeling the automatic process of artificial vision in order to provide solutions to the problem of inspecting specular surfaces. First of all, a model describing the image formation and the variables that take part in this process is presented.

An image *I* is defined as a two-dimensional representation provided by *F*. Let *F* a function that models a visual acquisition system, *VAS*. It includes all equipment and scene configuration to capture an image: lighting, positions, viewpoints, cameras, etc. Let ρ a vector made up of *scene magnitudes* that contribute to the formation of *I* Equation (1). Each vector ρ is an element of a representation space *P* related to optical magnitudes of the visual perception phenomenon.

(1) $$I\left(x,y\right):=F\left(\rho \right),\;\;\rho =\left(\rho_{1},\rho_{2},\rho_{3},...,\rho_{n}\right)\in P$$

The components $\rho_{i}$ of the vector of *scene magnitudes* are measurable physical values involved in the process of image formation. They could be, in practice: scale, viewpoint, light intensity, frequency, saturation, etc. Each component could be modeled as a function depending on three inputs Equation (2): the subject of interest, *m*, in the scene (e.g., the object to be inspected), the environment, *e*, in which the subject is placed and, finally, the camera, *c*, that captures images from the scene.

(2) $$\rho_{i}=\rho_{i}\left(m,e,c\right)$$

**Diagram 1:**
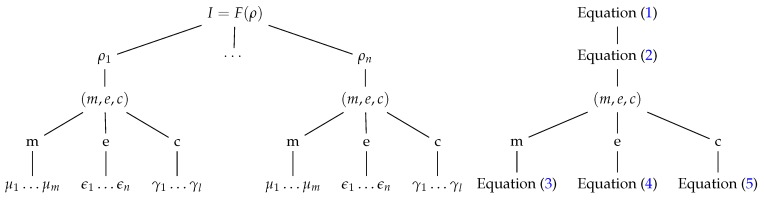
Levels of magnitudes involved in the formation of the image. The image function as the most abstract level on the top, then each scene magnitude, the three elements that composes ρ, and the individual measurements of each element on the bottom of the tree.

The contribution of each element (*m*, *e* and *c*) can be expressed as three vectors made up of magnitudes: $\mu_{i}$ related to the object Equation (3), $\epsilon_{i}$ related to the environment Equation (4) and $\gamma_{i}$ related to the camera Equation (5). Intensity and wavelength of light sources, medium of transmission, relative position between scene and vision device, are examples of environment variables $\epsilon_{i}$. Regarding the camera contribution $\gamma_{i}$, the variables are related to the sensor characteristics, optical and electronic elements: zoom, focus, diaphragm, size of the sensor, signal converters, etc. Finally, the reflectance, colour, shape, topography of the object are examples of object variables $\mu_{i}$. The values of each vector establish elements of the set *M* for the object, *E* for the environment and Γ for the camera. In Diagram 1 an outline of the magnitudes can be found.

(3) $$M=\left\{m_{0},m_{1},m_{2},...\right\},m_{i}=\left(\mu_{1},\mu_{2},...,\mu_{m}\right)$$

(4) $$E=\left\{e_{0},e_{1},e_{2},...\right\},e_{i}=\left(\epsilon_{1},\epsilon_{2},...,\epsilon_{n}\right)$$

(5) $$\Gamma =\left\{c_{0},c_{1},c_{2},...\right\},c_{i}=\left(\gamma_{1},\gamma_{2},...,\gamma_{l}\right)$$

**Figure 1:**
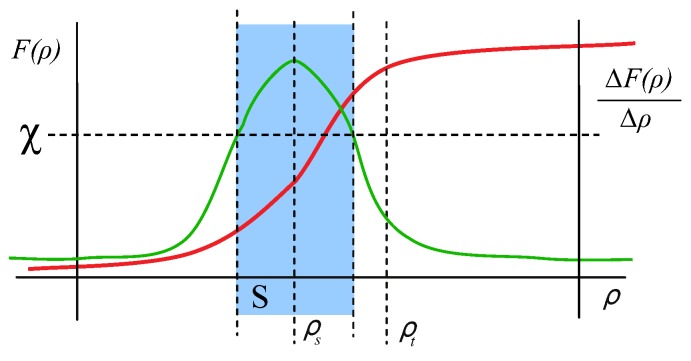
Calibration curve (**red**) and sensitivity (**green**) for the visual acquisition system (*VAS*) including *tuning* $\rho_{s}$ and *working point* $\rho_{t}$.

To define our model, it is worth to remember the sensitivity as an important static characteristic of a sensor. The sensitivity is the slope of the calibration curve (see Figure 1). First, we can define the calibration curve as the function that maps a physical scene magnitude and its representation in the image space. Depending on the camera parameters the calibration curve will have a different function for different scene magnitudes. For instance, a camera with large depth of field will have a smoother calibration curve for intensity measurement (along a larger set of scene magnitudes for intensity, the camera will be able to distinguish between them) than a camera with short depth of field which will have a very abrupt calibration curve for the same scene magnitude (only a small set of intensity values will be distinguishable). With this function, sensitivity indicates the detectable output change for the minimum change of the measured magnitude. As a naive example, the detectable output could be the ability to perceive two different color that are actually different in the real scene. If the color change is too small, the sensor would return the same value of intensity. In our case, the detectable output in F for the minimum change of the *scene magnitudes*:

(6) $$sensitivity=\frac{\Delta F(\rho )}{\Delta \rho }$$

Usually, the camera parameters are calibrated to a set of values $\gamma_{i}$ so that the sensitivity is optimized for all variables of ρ simultaneously with respect to a single metric (not necessarily maximized for each variable $\rho_{i}$). For example, a camera could be calibrated using a specific zoom, focus and sensor size optimizing the sensitivity of the system to perceive the colour of a subject in a wide range of object distances and viewpoints but the system is not optimized separately for distances or viewpoints. However, a given camera *c* has maximum sensitivity for a value of each $\rho_{i}$. For convenience, we are going to define the *tuning point* as the corresponding point $\rho_{s}$ in the *scene magnitudes* space *P* for each camera of the set Γ in which the sensitivity of the *VAS* is the optimum (see Figure 1) . The sensitivity decreases in general for values of ρ differently from $\rho_{s}$.

In the same way, since the *VAS* depends on the contribution of the environment *e*, for each value of the set *E*, the corresponding point $\rho_{t}$ in the *scene magnitudes* will be named as the *working point*. The detected output of this point is related to the sensitivity curve of the *VAS* for each of the magnitudes $\rho_{i}$ because it restricts the limits where the system can work. In average conditions or in simple approaches to vision problems, its effect is usually considered to be negligible because perception takes place close to $\rho_{s}$. This simplification is unacceptable in the case of adverse conditions as it occurs, for example, in dark environments, remote objects from the camera, or in the presence of specular objects that limit the capability of the *VAS* to perceive the scene.

**Figure 2:**
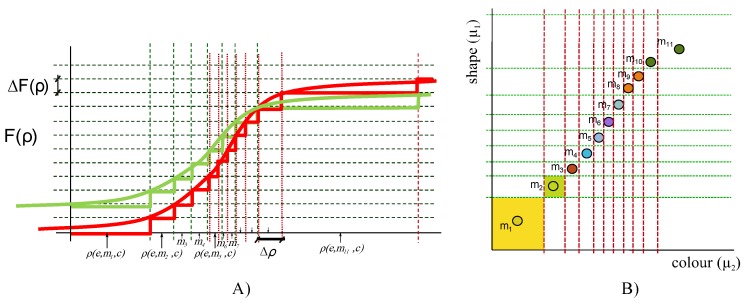
(**A**) Example of calibration curves for a *VAS* aimed to measure shape (**green**) and colour (**red**); (**B**) Measurement performed in *F* transformed to the object space *M* considering shape $\mu_{1}$ and colour $\mu_{2}$. In this example, the colour of the objects $m_{10}$ and $m_{11}$ can be distinguishable but their shape cannot.

Acquisition of the scene and representation on a plane carried out by *F* Equation (1) cause situations, related to vision in adverse conditions, where it is not possible to distinguish between the different scene magnitude vectors ρ that contribute to an image. The capacity to discern elements of *P* is related to the measurement in the image *I* of the magnitudes *m*, *e*, and/or *c*, that contribute to each of the components $\rho_{i}$. A specific application could be interested in knowing the intensity lighting of a scene from the environment *e*, or the focal length from the camera *c*, for calibration purposes. However, generally, the contribution of the object *m* to the image, and, therefore, its magnitudes $\mu_{i}$, is the aim of the measurement. For example, as we can see in Figure 2, a *VAS* used to perceive color ($\mu_{2}$) of objects is able to distinguish the different objects in the scene. However, if it is used to perceive shape ($\mu_{1}$) using the same camera (including all variables $\gamma_{i}$), neither objects $m_{8}$ and $m_{9}$ nor $m_{10}$ and $m_{11}$ could be distinguished each other (as we mentioned above, a given camera has maximum sensitivity for a specific value of each $\rho_{i}$). Hence, capacity to discern magnitudes that contribute to the vector ρ can be delimited to distinguish elements of set *M* in the image (e.g., the colour of a region of an object, or the shape of a surface, or both, etc.). It is important to understand that the scene magnitudes are continuous, not discrete. The representation in the Figure 2 shows a set of magnitudes that are distinguishable in the space of the image I=F(ρ), but it is an example of the certain magnitudes in the continuous space of magnitudes *P*.

An object could be distinguishable from another one in the *VAS* when they are distinguishable in the measurement performed in *F* (i.e., in the vertical axis of Figure 1 and Figure 2A). Let $\Omega_{m_{i}}$ be the set of objects that can be distinguished for an specific object $m_{i}$, and let χ the minimum difference perceptible by the system (sensitivity), then $\Omega_{m_{i}}$ could be established as:

(7) $$\begin{array}{c} \Omega_{m_{i}}=\left\{m_{j}\in M:\left(\exists \chi >0\right)\left[\left|F{(}^{m_{j}}\rho )-F{(}^{m_{i}}\rho )\right|\geq \chi \right]\right\} \end{array}$$

Figure 2B, shows the measurement performed in *F* analysed from the point of view of the object space *M* considering just colour and shape. Objects close to $m_{1}$, in the yellow area, are not distinguishable, for that object in this example. However, they are distinguishable for the object $m_{2}$ (light green area). Following this example, $m_{10}\in \Omega_{m_{9}}$ but $m_{10}\notin \Omega_{m_{11}}$, if the measurement performed in *F* takes into account both colour and shape.

A *VAS* has to deal with different situations that are a consequence of the subsets of *P* delimited by the problem to be solved (e.g., delimited by objects or by their characteristics to be analysed, or by environments, or by the cameras, etc.). A minimum value of sensitivity χ can be established in which any object is distinguishable from another considered in the acquisition (see Figure 1). The values of the vector of magnitudes ρ in which sensitivity is higher than a threshold χ conform the subset *S* of *P* defined by Equation (8).

(8) $$S=\left\{\begin{array}{c} \rho_{i}:\left(\exists \chi >0\right)\left(\forall m_{k},\forall m_{j}\in M\right)\left[\frac{\left|F\left(\rho_{i}\right)-F\left(\rho_{i-1}\right)\right|}{\Delta \rho }\geq \chi \to m_{j}\in \Omega_{m_{k}}\right] \end{array}\right\}$$

There are no perception difficulties for situations of the subset S⊂P. They involve values of environment and camera, in addition to characteristics of objects, that make up magnitudes ρ of the set *S* Equation (8). In other words, it is possible to distinguish the images of two different objects ($m_{i}$ and $m_{j}$ in Equation (9)) from the acquisition performed by *F* if the *VAS* is working on points of *S* (${}^{m_{i}}\rho$ denote a scene magnitude vector whose component *m* is the object $m_{i}$).

(9) $$\begin{array}{c} \begin{array}{c} \left(\exists^{m_{i}}\rho {,}^{m_{j}}\rho \in S\right)\left(\forall m_{i},m_{j}\in M\right)\left[\left(m_{i}\neq m_{j}\right)\to \left|F{(}^{m_{i}}\rho )-F{(}^{m_{j}}\rho )\right|>0\right] \end{array} \end{array}$$

**Diagram 2:**
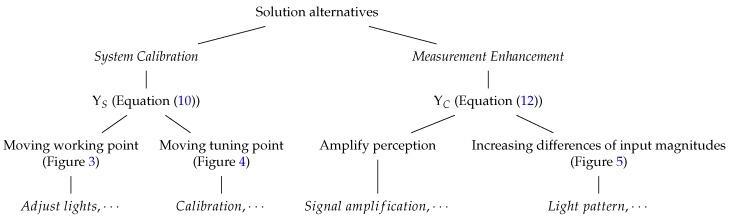
Diagram with the different alternatives to improve the system perception.

Vision systems working on *scene magnitudes* of the complementary of *S*, $S^{c}$, (P\S) present the aforementioned adverse conditions to distinguish characteristics of objects from images. Hence, solutions have to be provided to achieve distinct images from different objects that the camera perceives as the same one. These solutions should be able to compensate the low sensitivity in $S^{c}$ (sensitivity less than χ in Figure 1). Among the three variables that provide values to the *scene magnitudes*, object is a constant due to them being the subject of interest. However, environment conditions and camera characteristics could be modified to set up *scene magnitudes* ρ of the subset *S*. Thus, the *VAS* is going to be able to have different images from different objects in the scene in order to distinguish them Equation (9). For this purpose, we propose two complementary alternatives (Diagram 2):

- *System Calibration* (calibrating the system). This alternative tries to minimize the distance between the *working point* $\rho_{t}$ and the *tuning point* $\rho_{s}$.
- *Measurement Enhancement* (conditioning the measurement). Enhance the target measurements or parameters. This alternative can be considered as conditioning the measurement performed by the *VAS*.

**Figure 3:**
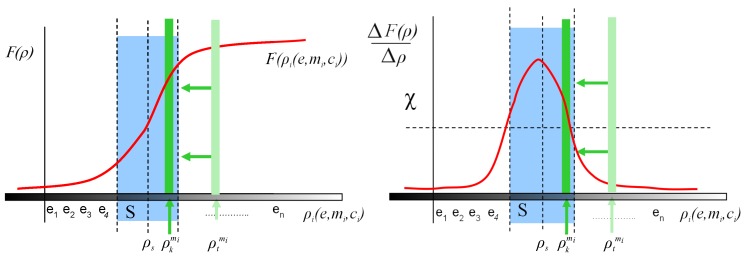
Outline for a *System Calibration* $\Upsilon_{S}$ shifting the *working point* to *S*. The initial *working point* $\rho_{t}^{m_{i}}$ (**light green**) has been moved to $\rho_{k}^{m_{i}}$ (**dark green**) after transformation is applied. The new *working point* $\rho_{k}^{m_{i}}$ is close enough to the *tuning point* $\rho_{s}$.

**Figure 4:**
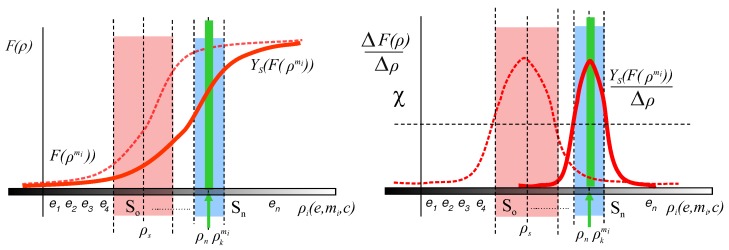
Outline for a transformation $\Upsilon_{S}$ shifting the *tuning point* to the *working point* $\rho_{k}^{m_{i}}$. Dotted line curve and light red area represents the old calibration and $S_{o}$ respectively. Red line curve and blue area represent the new calibration curve and the new $S_{n}$. The old *tuning point* $\rho_{s}$ has been consequently moved to the new one $\rho_{n}$ after transformation is applied.

*System Calibration* consists of shifting one of the points so that the *working point* be an element of the set *S*. The goal could be generating a new image of the object $m_{i}$ by a transformation $\Upsilon_{S}$ Equation (10) in which the *working point* ($\rho_{k}^{m_{i}}$) be close enough to the *tuning point*. Do not confuse calibrating the system with calibrating the camera. The alternative presented here could be calibrating the sensor as well as changing other parameters of environment or object of interest. Hence, to carry out this alternative it is necessary to adjust the environment to shift the *working point*, for example moving the object closer to the camera, adjusting lighting conditions, etc. (Figure 3 shows an outline of this process). On the other hand, in order to shift the *tuning point* (see Figure 4), the camera could be recalibrated or new acquisition equipment can be used (traditionally this is done by replacing the camera with a more suitable one). In this case, subset *S* of *P* changes for the new camera from $S_{o}$ to $S_{n}$.

(10) $$\begin{array}{c} \left(\exists \Upsilon_{S}\right)\left(\exists \rho_{k}^{m_{i}}\in S\right)\left(\forall \rho^{m_{i}}\in P\right)\left[F\left(\rho_{k}^{m_{i}}\right)=\Upsilon_{S}\left(F(\rho^{m_{i}})\right)\right] \end{array}$$

For the second alternative, *Measurement Enhancement* aims to directly influence the sensitivity curve of the acquisition system. In a nutshell, it tries to somehow highlight or enhance the parameters ρ which want to be perceived. It can be carried out by means of two new alternatives. First, the output signal of the perception system can be amplified. That is, the classic conception of measurement system amplification at the signal conditioning step. Limitations of this technique are related to increasing the amplitude of the signal in ranges of minimal sensitivity (both minimum and maximum of the range of the sensor because the contribution of the object in the output signal is insignificant, the signal-to-noise ratio is very low). In this way, acquisition system improvements are limited because they are only applied in ranges of intermediate sensitivity.

**Figure 5:**
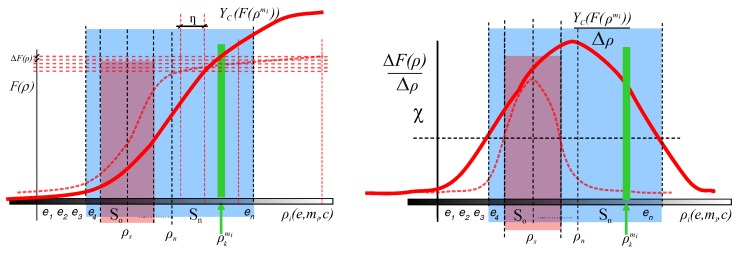
Outline for a transformation $\Upsilon_{C}$ able to increase differences of input magnitudes values until the change is perceptible in the output *F*. Dotted line curve and light red area represents the old calibration and $S_{o}$ respectively of the *VAS*. Red line curve and blue area represent the new calibration curve and the new $S_{n}$. The vertical dotted red lines represent values of the set *A* that allow perceptible changes in the output (horizontal dotted red lines).

The other alternative of enhancing the target measurement is increasing the differences of the values of the input magnitudes. This is to operate with large differences (η) of the input (ρ) to increase the differences of the output *F* for different objects ($m_{i}$ and $m_{j}$) until the differences are perceptible at the output ($m_{j}\in \Omega_{m_{i}}$ ). Figure 5 schematically shows this concept. Elements used as input magnitudes of the set *P* to get large differences of the input make up the subset *A* in Equation (11). The goal is to reduce the number of possibilities that the *VAS* has to deal with; for example, restricting camera positions, viewpoints or lighting characteristics (in this paper, a lighting pattern has been used to inspect specular surfaces).

(11) $$A=\left\{\begin{array}{c} \rho_{k}^{m_{i}}:\left(\exists \eta >0\right)\left(\forall \rho_{l}^{m_{j}}\in P\right)\left[[\left(m_{i}\neq m_{k}\right)\wedge \left|\rho_{k}^{m_{i}}-\rho_{l}^{m_{j}}\right|\geq \eta ]\to m_{j}\in \Omega_{m_{i}}\right] \end{array}\right\}$$

We model the transformation able to increase the differences of the values of the input magnitudes as $\Upsilon_{C}$:

(12) $$\begin{array}{c} \left(\exists \Upsilon_{C}\right)\left(\exists \rho_{k}^{m_{i}}\in A\right)\left(\forall \rho^{m_{i}}\in P\right)\left[F\left(\rho_{k}^{m_{i}}\right)=\Upsilon_{S}\left(F\left(\rho^{m_{i}}\right)\right)\right] \end{array}$$

The techniques are not exclusive and can be used together for designing vision system in which images of different objects can be distinguished. An example of the use could be a system which needs to perceive two colliding objects separately through a sensor with a fisheye lens. First, a $\Upsilon_{S}$ transformation by means of calibrating the camera to reduce the distortion could be applied. After, $\Upsilon_{C}$ could mean to colorize the objects to enlarge the perceived difference between them.

## 3. Method for Inspecting Specular Surfaces

In this section, we are going to use the previous model to specify a method for inspecting specular surfaces. The objective of an automated visual inspection system, *AVI*, aims to determine if a product differs from the manufacturer’s specifications. This implies the *AVI* has to measure the object magnitudes in the scene in order to compare them with values of the magnitudes established in the design step of the product (e.g., reflectance, colour, shape, topography).

Two sets of objects are considered for modelling the *AVI*: $M_{P}$ and $M_{I}$. The first one is composed of objects that are made up by magnitudes $\mu_{i}$ defined in the manufacturing specifications. The set $M_{I}$ is composed by objects to be inspected looking for any deviation from objects of the set $M_{P}$. These deviations cause, depending on the magnitude, different defects: morphological, chromatic, topographic defects, etc. The union of $M_{P}$ and $M_{I}$ is the subset $M_{S}=\left\{M_{P}\cup M_{I}\right\}\subset M$ Equation (3) of the possible objects that the inspection system must consider.

The inspection goal could be modelled as in Equation (13). The *AVI* has to decide whether an object $m_{i}$ of the set $M_{I}$ could be distinguished (9) from an object $m_{j}$ of the set $M_{P}$ if any of the magnitudes $\mu_{i}^{m_{j}}$ differ in some value η from the original $\mu_{i}^{m_{k}}$. The object $m_{j}$ is considered the object model of $m_{i}$ and contains the manufacturing specifications.

(13) $$\begin{array}{c} \left(\exists i\right)\left(\exists \eta >0\right)\left(\forall m_{j}\in M_{I}\right)\left(\forall m_{k}\in M_{P}\right)\left[\left|\mu_{i}^{m{}_{j}}-\mu_{i}^{m{}_{k}}\right|\geq \eta \to m_{j}\in \Omega \left(m_{k}\right)\right] \end{array}$$

In order for the deviations (η) of the object magnitudes $\mu_{i}^{m_{j}}$ from $\mu_{i}^{m_{k}}$ to be detected in the image Equation (13), contributions from the environment and the camera to ρ must allow a suitable sensitivity to the *AVI*.

Since it was previously shown that the sensitivity of the *VAS* (*AVI* in this specific case) is optimized simultaneously for all variables of ρ, *AVI* sensitivity is not necessarily maximized for each variable $\rho_{i}$. It is not maximized for each object *m* and each of its variables $\mu_{i}$. Moreover, generally an *AVI* is designed for performing a measurement of a subset of the object magnitudes $\mu_{i}$ in the image (i.e., colour, shape). Therefore, the sensitivity of the *AVI* can be very low for some of the magnitudes $\mu_{i}$ to be measured. That is, it is possible that the perception be suitable for measuring the surface colour or shape but it could not be suitable for both together. In other words, the intended measurement determines the perception capacity of the *AVI*. Calibration parameters or environment conditions must be adjusted to adequately perceive magnitudes $\mu_{i}$. This process requires great knowledge of the problem and accuracy for the solution.

For specular surfaces, the difficulty to perceive in *scene magnitudes* of $S^{c}$ is a consequence of the surface reflectance. The environment conditions produce an effect in the perception of the object being more important than other types of surfaces (e.g., Lambertian surfaces). For example, if it is considered that the calibration parameters $\gamma_{i}$ are the same for two different images, the difficulty to perceive with different environment conditions is given by:

- The mirror itself: a given camera can be confused so that it cannot distinguish between the environment and the object. The spatial modulation of the environment contribution creates the illusion of the objects in a scene.
- The lighting of the environment can cause shine on the surface and confuse the two images with different objects. For example, the image formed by a grey surface with a high reflection coefficient illuminated by white lights can be confused with the image of a lighter surface object.

The specular reflection causes the *working point* $\rho_{t}$ be easily located at the limit of the range of the *VAS*, at the maximum value of ρ that can be measured. Sensitivity is very low for the perception of objects in these conditions because specularity saturates the camera sensor.

The viable solution for compensating the lack of sensitivity produced in the ranges of the *scene magnitudes* ρ (in order to obtain an improved image) is related to the *System Calibration* that performs the perception system (see Section 2). It is necessary to increase the differences in the values of the input magnitudes, $\Delta_{\rho }$, to raise the differences of the output magnitude until they can be measured (Equation (12)).

In other words, using the resolution (another important sensor characteristic that indicates the smallest change in the magnitude being measured that the sensor can detect, e.g., the smallest feature size of an object or the smallest change in colour that the *VAS* can distinguish), since the resolution in those ranges is very low, it is necessary to force large differences at the input. Thus, it is necessary to work using a subset of *scene magnitudes* A⊂P in order the perception will be suitable in the points around the *tuning point* and it will be facilitated in the ranges of sensor saturation. Differences in *F* will enable the perception among objects using the set $S_{A}$ in Equation (14). The elements do not necessarily correspond to those of subset *S* Equation (8).

(14) $$S_{A}=\left\{\begin{array}{c} \rho_{i}\in A:\left(\exists \chi >0\right)\left(\forall m_{i}\in M_{P}\right)\left(\forall m_{j}\in M_{I}\right)\left[\frac{\Delta F(\rho_{i})}{\Delta \rho }\geq \chi \to m_{j}\in \Omega_{m_{i}}\right] \end{array}\right\}$$

**Figure 6:**
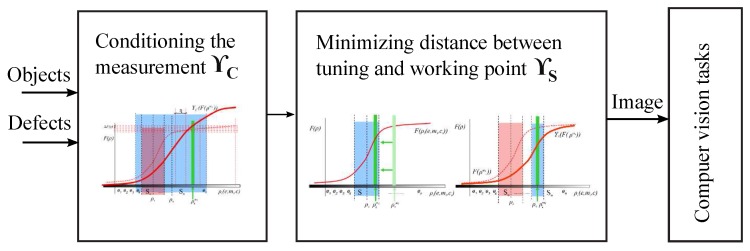
Method for inspecting specular surfaces. According to objects and defects, transformations $\Upsilon_{S}$ and $\Upsilon_{C}$ are selected to provide an image in which defects can be detected using computer vision techniques.

In addition, if the *Measurement Enhancement* is not sufficient to discern the defects in the inspection, the distance between the *working point* $\rho_{t}$ and the *tuning point* $\rho_{s}$ must be minimized using the Equation (10). In this case, minimization is performed on the input magnitudes of the set *A*. Therefore, the transformation $\Upsilon_{S}$ operates with the values of the input magnitudes $S_{A}$ Equation (14). In consequence, Equation (15) models the proposed solution for inspecting specular surfaces combining the two proposed transformations $\Upsilon_{S}$ and $\Upsilon_{C}$ according to the objects to be inspected and the possible defects to be detected (see Figure 6).

(15) $$\begin{array}{c} \left(\exists \Upsilon_{C}\right)\left(\exists \Upsilon_{S}\right)\left(\exists \chi ,\eta >0\right)\left(\exists \rho_{k}^{m_{i}},\rho_{l}^{m_{l}}\in A\subset P\right)\\ \left(\exists \rho_{o}^{m_{i}},\rho_{q}^{m_{j}}\in S_{A}\right)\left(\forall \rho^{m_{i}},\rho^{m_{j}}\in P\right)\\ F\left(\rho_{k}^{m_{i}}\right)=\Upsilon_{C}\left(F\left(\rho^{m_{i}}\right)\right),F\left(\rho_{l}^{m_{j}}\right)=\Upsilon_{C}\left(F\left(\rho^{m_{j}}\right)\right)\\ F\left(\rho_{o}^{m_{i}}\right)=\Upsilon_{S}\left(F\left(\rho_{k}^{m_{i}}\right)\right),F\left(\rho_{q}^{m_{i}}\right)=\Upsilon_{S}\left(F\left(\rho_{l}^{m_{j}}\right)\right)\\ \left(m_{i}\neq m_{j}\right)\wedge \left|\rho_{k}^{m_{i}}-\rho_{l}^{m_{j}}\right|\geq \eta \to \left|F\left(\rho_{o}^{m_{i}}\right)-F\left(\rho_{q}^{m_{j}}\right)\right|\geq \chi \end{array}$$

## 4. AVI Method Controlling Environmental Parameters

As it was previously shown, increasing the differences of the input magnitude values $\Delta_{\rho }$ can be performed by means of the control of the environmental conditions or of the camera parameters. These are two input parameters of the components $\rho_{i}$ Equation (2) that contribute to the formation of the image I Equation (1) and are variables that can be affected by the system. The third input parameter, the object, is considered as a constant because it is the object to be inspected.

**Figure 7:**
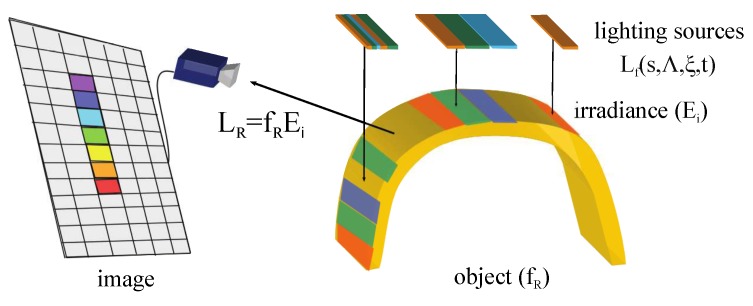
Parameters involved in the scene radiance $L_{R}$: object reflectance ($f_{R}$) and irradiance (*E*) incident on the surface.

It is known that the camera, as a photoelectric transducer, provides a measurement related to the scene radiance (see Figure 7). It is a function of environment and object magnitudes. The contribution of interest to the radiance is the radiance coming from the inspected object. This radiance, $L_{R}$, is related to the object reflectance, $f_{R}$ [66] and the irradiance *E* incident on the surface according to Equation (16).

(16) $$L_{R}=f_{R}Ei$$

Reflectance $f_{R}$ contributes the necessary information about the behaviour of the light interacting surface of the object. Irradiance, the second factor of Equation (16), is related to the environment variables, in fact, to be precise, to the electromagnetic radiation that reaches the surface of the object. These magnitudes affect the contribution of the object in the camera.

Controlling the luminous energy of the environment, a function of the lighting sources and the transmission modulations, is a way of working directly with input magnitudes of the system. Without considering any other perception characteristics, irradiance is the key. Structuring the energy that reaches the object is a method of affecting the environment or the object. It enables areas of the surface of the object to be isolated, the contrast in the camera to be increased or decreased, etc. Thus, this variable allow us to force large differences at the input to perceive changes in the output image.

*Measurement Enhancement* is the task of the transformation $\Upsilon_{C}$ Equation (15). In this paper, we propose a instance $\Upsilon_{\Phi }$ of $\Upsilon_{C}$ focused on lighting conditions of the environment. The transformation $\Upsilon_{\Phi }$ structures the lighting in order to establish regions on the object radiated by different spectral powers Φ. Areas of different radiances are formed in this way. The regions can be formed by means of spatial modulation, forming a grid. Also, a sequence of lightings using temporal modulation or a combination of both can be used. The regions contribute with different radiances to the input of the camera establishing independent areas in the image. The increasing of the differences at the input of the system is produced in space or time domain Equation (12).

In this paper, the transformation $\Upsilon_{\Phi }$ is carried out by means of spatial modulation (space domain). The different areas in the image are formed with characteristics proportional to the irradiance *E*. The object irradiance *E* actively affects the perception process. The environment parameters are established so that great lighting gradients on the object are formed. The projection of the radiance *L*, which arrives at the optics, forms an image with regions. The projection of the radiance L, which arrives at the optics, forms an image with regions. The gradient of the image is a function of the one generated on the object. The greater the gradient of the pattern, the greater the gradient formed on the photodetectors. Then, a larger difference among adjacent photodetectors is obtained. Therefore, each photodetector has a spectral power associated in an instant (that is the function of irradiance and object characteristics).

Structuring the lighting enables the projection of a pattern on the surface object. This pattern is deformed by the object characteristics. It may be considered that the irradiance *E* is modulated by the object. Then, any other object modulates the generated pattern in a different way, and, therefore, modulates the spectral power, which is received in the space or the time by each photodetector, in a different way (in terms of inspection systems, any object with defects will modulate the generated pattern in a different way than the same object without defects). Controlling the input values of the perception system, for each photodetector, enables a reduction in the elements of the set of *scene magnitudes* that the system has to deal with. In addition, the pattern has to be configured so that the differences of the output are perceptible according to the magnitudes of the object (shape, colour, topology, etc.) to be perceived.

Control of energy that reaches the surface of the object is needed in order to design a certain pattern. The task depends on the number of environment lights, the spatial distribution of lighting, the wavelengths that conform each of the sources, the time, the modulations of transmission, etc. Moreover, the pattern of spectral power Φ could be different in order to inspect a specific magnitude $\mu_{i}$ of the object *m*. Then to this purpose, the transformation $\Upsilon_{\Phi }$ will determine the spectral power Φ∈P as a function of $\mu_{i}\in U$ and m∈M:

(17) $$\Upsilon_{\Phi }:M\times U\to P$$

**Figure 8:**
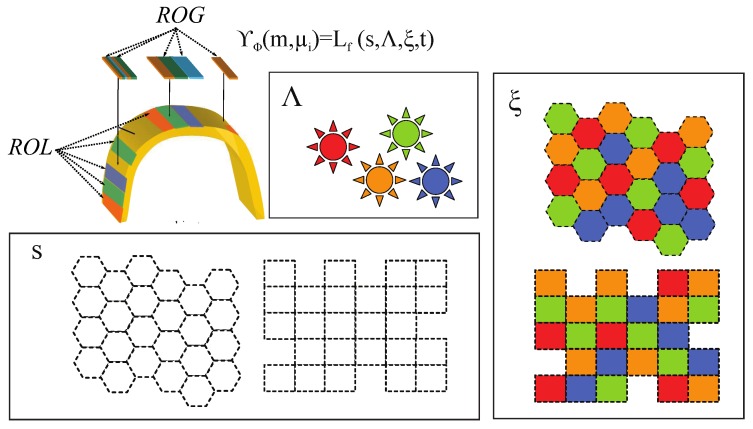
Parameters *s*, Δ, ξ of the transformation $\Upsilon_{\Phi }$.

For practical considerations, it is interesting that the function of energy establishing the spectral power Φ is established in terms of the field radiance $L_{f}$ by considering four parameters: s∈S, Δ∈D, ξ∈X and t∈R (see Figure 8).

(18) $$L_{f}:S\times D\times X\times R\to P$$

Hence, the transformation $\Upsilon_{\Phi }$ could be defined by:

(19) $$\begin{array}{c} \Upsilon_{\Phi }\left(m,\mu_{i}\right):=L_{f}\left(s,\Delta ,\xi ,t\right) \end{array}$$

The regions of lighting ROL on the surface of the object can be determined from the parameter *s*. It is a function that establishes the morphology of the regions of the pattern formed on the surface. Parameter Δ determines the set of lighting characteristics that radiates each of the established regions ROL. The function ξ determines the spatial configuration of energy reached by the object. The task of the function is to distribute the lighting characteristics of the set Δ over each of the regions determined by *s*. Finally, the function depends on time *t*. If the structured lighting is temporal, it is necessary to generate a sequence of patterns. Moreover, for practical reasons, we define ROG as the regions of lighting on the source. In the same way as ROL, the ROG is the morphology of the regions of the pattern conformed on the lighting source, in this case (see Figure 8).

## 5. Experiments

In this section, experiments performed by simulations to validate the model to deal with specular surfaces are presented. Specifically, the objective is to prove whether the transformation $\Upsilon_{\Phi }$ is able to compensate for the lack of sensitivity that takes place in certain values of the magnitudes of the scene. In other words, probing whether the transformation *Measurement Enhancement*, by actively controlling the lightning pattern, is able to detect object characteristics (e.g., defects in visual inspection) that are not detected in other conditions. The experiments are mainly based on the scale of perception and the point of view as magnitudes of the scene.

We propose the use of simulation based on virtual imaging as an efficient way to perform a large battery of tests (different environment conditions and camera parameters) as a previous step of experimentation in real manufacturing environments, more complex in terms of instrumentation, and thus more expensive. Then, in this section, experiments performed by simulations to validate the model to deal with specular surfaces are presented.

### 5.1. Experimental Setup

An extensive explanation of the experimental setup is shown. It integrates the simulator, the subject of interest, and the environmental parameters that allow to design the lighting patterns.

#### 5.1.1. Simulator

A simulator has been implemented considering the model presented in Section 2. It is able to synthesize images by F(ρ) Equation (1). The components of $\rho_{i}$ Equation (2) of the *scene magnitudes* (scale, viewpoint, light intensity, etc.) are modelled by the characteristics of the subject of interest, *m*, the environment, *e*, and, finally, the camera, *c* (see Diagram 1).

According to the characteristics $\mu_{i}$ of the subject of interest *m*, the simulator is basically interested in the reflection characteristics of the surfaces. The light reflection on an object depends on the atomic nature of the surface material and on the scattering and dispersion that takes place when the light contacts it. Consequently, in order to determine the reflected flow densities, the spatial configuration of the surface $\mu_{S}$ and its electromagnetic properties, which determine the type of material, are taken into account. These properties provide the refraction index $\mu_{n}$ of the surface, which represents the macroscopic optical properties required.

Regarding the environment *e*, the problem of specifying it is limited to the specific application of inspection. Therefore, in the process of transmitting the light signal from generating sources to the image, the different lighting sources are considered together with the modulations that the signal experiences, with the exception of those referring to the object to be inspected and those of the acquisition system. As environment variables, a distinction will be made between those related to lighting sources (that make up the fundamental element in the environment magnitudes) and the relative positions of the different elements involved in the scene. In order to carry out the study of local light reflection, the Bidirectional Reflectance Distribution Function (BRDF) presented by Cook and Torrance [67] has been used because different studies show its great capacity to adapt to reflectance extracted from objects [68].

Finally, variables related to the characteristics of the perception system include both the functions that define the optics and those related to the sensor itself. The aim of this research is to study the influence of environmental conditioning on the perception of the object. Then, controlled experimentation aimed to measure the radiance coming from the subject of interest is performed. Therefore, an ideal perception system is considered (images without noise, constant sensitivity for all spectral powers, constant diaphragm, etc.). It influences the radiometric measurement only with geometric variables like the focal distance and the size of the sensor. The other variables have been set to be constant.

#### 5.1.2. Subject of Interest

Plane objects are considered in the experiments. They permit a simple control of the angle formed by the elements involved in the scene on all regions of the object. Control is necessary due to that fact that one of the aims of experimentation is to carry out a thorough test of the viewpoint.

The set $M_{P}$ of the objects defined in the manufacturing specifications is made up of planes of 12 mm × 12 mm. The function $\mu_{S}$ establishes the points in a coordinate system that is local to the object independently of microscopic shape. The surface roughness is calculated by means of the Beckmann distribution model [69] by coherence with the BRDF model used. The root mean square is assigned to 0.1 (rms, or m in [67]). According to electromagnetic characteristics, there are two types of plane: dielectric and metallic. Dielectric planes have a refraction index $\mu_{n}$ of 1.6 and metallic ones (base on chromium) have a refraction index $\mu_{n}$ of 2.8 and an extinction index $\mu_{n_{i}}$ of 3.2 using the Fresnel equations. Also, other variables of the Cook-Torrance model such as a specularity coefficient (*s* in [67]) of 0.75 and a constant diffuse component ($R_{s}$) associated to a RGB of (0.6,0.6,0.6) have been considered.

**Figure 9:**
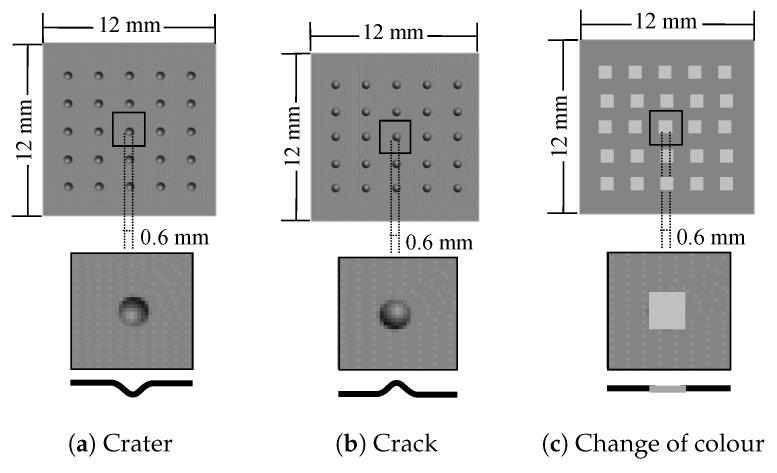
Defects considered in the experiments.

The set $M_{P}$ to be inspected is made up of objects with the same characteristics of $M_{P}$ but including defects. Three types of defects have been distributed over these planes (see Figure 9): two changes in topography (a 0.6 mm-diameter crack or crater in Figure 9a,b and a change in colour (an area of 0.6 mm × 0.6 mm in Figure 9c). In this last case, a constant diffuse component $R_{s}$ associated to a RGB of (0.4,0.4,0.4) on a surface measuring 0.6 mm × 0.6 mm is established. In total, for each plane in $M_{P}$, 3 planes (1 per type of defect) with 25 defects have been considered in $M_{P}$. Table 1 summarizes the data used in the experiments.

#### 5.1.3. Environment

Environment characteristics play an important role in validating the transformation $\Upsilon_{\Phi }$ Equation (19). Specifically, the goal is to study the transformation that configures the lighting of the scene to establish regions on the object surface radiated by different spectral powers by means of spatial modulation. Therefore, we have considered different spatial configurations of the energy that reaches the object surface according to different gradients by considering different *s*, Δ and ξ.

Since the objects are planes, the regions of lighting ROL formed by *s* are established on a plane. In the experiments, the parameters are established from the lighting source domain using the ROG (see Figure 8). The set of lights conform a grid so that the whole lighting extension can be defined for different conditions. Different areas of the regions of grid ROG are considered 0.1 mm × 0.1 mm, 0.2 mm × 0.2 mm, 0.3 mm × 0.3 mm and 0.6 mm × 0.6 mm to determine the influence of the size of the regions inside the areas of defects. Then, the ratios between the size of the region and the size of the defect in one of the dimensions (0.6 mm) are: 6 (0.6/0.1), 3 (0.6/0.2), 2 (0.6/0.3) and 1 (0.6/0.6).

The set Δ Equation (20) is made up of lighting sources using the same characteristics (polarization, power, etc.) except the wavelengths, $\delta_{i}$, to conform different spectral powers, $\Phi_{\delta }$. The wavelengths used are from the visible electromagnetic spectrum. Also, lighting sources only radiate for specific wavelengths (monochromatic lights).

(20) $$\Delta =\left\{\Phi_{\delta_{0}},\Phi_{\delta_{1}},\Phi_{\delta_{2}},...\right\}$$

**Figure 10:**
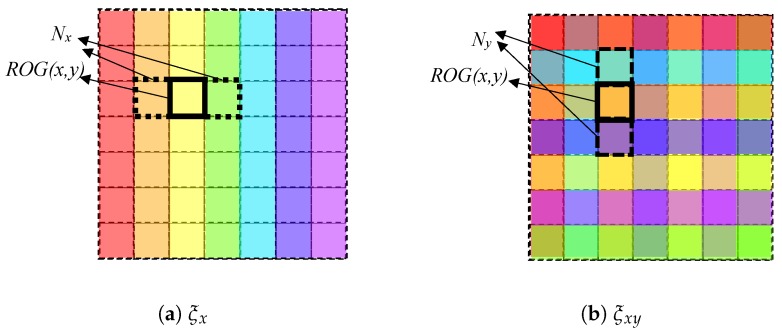
Spatial distribution considered for function ξ in the experiments.

Finally, the function ξ determines the spatial configuration of energy emitted by the lighting. The lighting characteristics of the set Δ are distributed over each of the regions ROL determined by *s* establishing different gradients: spatial and amplitude. In the experiments, for practical considerations, the ROG composed of a squared grid has been used (Figure 10).

**Figure 11:**
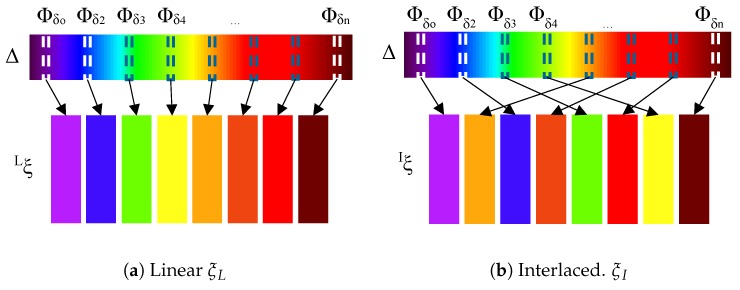
Amplitude distribution considered for function ξ in the experiments.

In this paper, four different configurations of lighting are considered: two for spatial gradients ($\xi_{x}$ and $\xi_{xy}$) and two for amplitude gradients ($\xi_{L}$ and $\xi_{I}$). Regarding the former two, the function ξ establishes spectral powers of Δ Equation (20) into two different spatial distributions. Specifically, in the experiments the spatial gradient established by ξ is organized in one direction, $\xi_{x}$, and in two directions, $\xi_{xy}$, (see Figure 11). Let ROG(x,y) be the region of column *x* and row *y* of the lighting grid and let $N_{x}$, $N_{y}$ be the column and row of neighbouring regions of the grid. A function ξ will be defined as $\xi_{x}$ if it only sets up different lighting characteristics in adjacent positions of an axis of the grid (Figure 10a) and the same ones in adjacent positions of the other axis of the grid. Then, any region in the grid ROG is assigned an element of the set Δ such that:

(21) $$\begin{array}{c} \xi_{x}\left(ROG(x,y),\Delta \right)=\Phi_{\delta_{i}}\in \Delta :\xi (N_{y}\left(ROG(x,y)\right),\Delta )=\Phi_{\delta_{i}}\wedge \xi (N_{x}\left(ROG(x,y)\right),\Delta )\neq \Phi_{\delta_{i}} \end{array}$$

For the second spatial gradient, a function ξ will be defined as $\xi_{xy}$ if sets up different lighting characteristics in all adjacent positions of a region of the grid (Figure 10b). Any region in ROG is assigned to an element of the set Δ Equation (20) such that:

(22) $$\begin{array}{c} \xi_{xy}\left(ROG(x,y),\Delta \right)=\Phi_{\delta_{i}}\in \Delta :\xi \left(N_{y}\left(ROG(x,y)\right),\Delta \right)\neq \Phi_{\delta_{i}}\wedge \xi \left(N_{x}\left(ROG(x,y)\right),\Delta \right)\neq \Phi_{\delta_{i}} \end{array}$$

Regarding the amplitude gradients, two configurations are also considered for ξ: *Linear*, $\xi_{L}$, and *Interlaced*, $\xi_{I}$. A function ξ will be defined as $\xi_{L}$ if sets up lighting characteristics with close wavelengths in adjacent positions *N* of the regions of the lighting grid ROG (see Figure 11a) whereas the *Interlaced*, $\xi_{I}$, configuration maximize the differences among wavelengths in these positions (see Figure 11b). Then, in case of $\xi_{L}$, any region in ROG is assigned to an element of the set Δ Equation (20) such that:

(23) $$\begin{array}{c} \xi_{L}\left(ROG(x,y),\Delta \right)=\underset{\Phi_{\delta_{i}}}{\mathrm{argmin}}(\xi \left(N\left(ROG(x,y)\right),\Delta \right)-\Phi_{\delta_{i}})\;\mathrm{subject}\;\mathrm{to}\;\sum \xi \left(N\left(ROG\left(x,y\right)\right),\Delta \right)-\Phi_{\delta_{i}}>0 \end{array}$$

In case of any function that is considered as an *Interlaced* function, $\xi_{I}$, the ROG is assigned such that:

(24) $$\begin{array}{c} \xi_{I}\left(ROG\left(x,y\right),\Delta \right)=\underset{\Phi_{\delta_{i}}}{\mathrm{argmax}}(\xi \left(N\left(ROG(x,y)\right),\Delta \right)-\Phi_{\delta_{i}}) \end{array}$$

Different transformations $\Upsilon_{\Phi }$ are formed using combinations of functions ξ accomplishing different spatial and amplitude gradients: *Linear X* ($\xi_{L,x}$), *Interlaced X* ($\xi_{I,x}$), *Linear XY* ($\xi_{L,xy}$), and *Interlaced XY* ($\xi_{I,xy}$) to carry out the experiments (see Table 2).

The function *Linear X* ($\xi_{L,x}$) is made up by using a function meeting a spatial gradient in one axis of the grid Equation (21) and an amplitude gradient formed by *Linear* configuration Equation (23). This function uses an ordered sequence $\Delta^{L}$ of the set Δ. Let $\Phi_{\delta_{i}}$ be an element of Δ that radiates energy with the wavelenght $\delta_{i}$. Let $\delta^{i}$ and $\delta^{e}$ be the minimum and maximum values and let $\delta^{n}$ be the number of wavelengths considered in the sequence, then $\Delta^{L}$ is:

(25) $$\begin{array}{c} \Delta^{L}=\left[\Phi_{\delta_{0}},\Phi_{\delta_{1}},\Phi_{\delta_{2}},...,\Phi_{\delta_{n-1}}\right]:n\geq 2,\delta^{e}\geq \delta^{i},\delta_{i}=\delta^{i}+mod\left(i,\delta^{n}\right)\left(\frac{\delta^{e}-\delta^{i}}{\delta^{n-1}}\right) \end{array}$$

An element of the sequence $\Delta^{L}$ is assigned to the region (*x*,*y*) of the grid lighting ROG by the function $\xi^{x}$:

(26) $$\xi^{x}\left(ROG\left(x,y\right),\Delta^{L}\right)=\Delta^{L}\left(mod(x,n)\right)$$

The differences of wavelengths among neighbouring regions are constant in one of the axes of the grid.

The function *Interlaced X*($\xi_{I,xy}$) is made up by a function that accomplishes a spatial gradient in one axis of the grid Equation (21) and an amplitude gradient formed by the ’*Interlaced*’ configuration Equation (24). In this paper, this function uses an ordered sequence $\Delta^{E}$ of the set Δ in which a maximum difference of the wavelengths between adjacent positions is established. Let $\Delta_{t}$ and $\Delta_{b}$ be the top half and bottom half of $\Delta^{L}$ Equation (25).

(27) $$\begin{array}{c} \Delta_{t}=\left[\Phi_{\delta_{0}},\Phi_{\delta_{1}},...,\Phi_{\delta_{i}-1}\right],\\ \Delta_{b}=\left[\Phi_{\delta_{i}},\Phi_{\delta_{i}+1},...,\Phi_{\delta_{n}-1}\right]:\\ i=\left\lceil n/2\right\rceil ,\Delta_{t}\cup \Delta_{b}=\Delta^{L} \end{array}$$

Then, the sequence $\Delta^{E}$ combines $\Delta_{t}$ and $\Delta_{b}$ in an interlaced manner:

(28) $$\begin{array}{c} \Delta^{E}=\left[\delta_{0},\delta_{1},...,\delta_{n-1}\right]:\\ \delta_{i}=\left\{\begin{array}{cc} \Delta_{t}\left(\left\lfloor i/2\right\rfloor \right) & \mathrm{if}\;(i\mathrm{mod}2)=0\\ \Delta_{b}\left(\left\lfloor i/2\right\rfloor \right) & \mathrm{if}\;(i\mathrm{mod}2)=1 \end{array}\right] \end{array}$$

An element of the sequence $\Delta^{E}$ is assigned to the region (x,y) of the grid by the function $\xi^{x}$ Equation (26).

The function ’*Linear XY*’ accomplish a spatial gradient in two axes of the grid Equation (22) and an amplitude gradient formed by the ’*Linear*’ configuration Equation (23). This function uses the ordered sequence $\Delta^{L}$ Equation (25) of the set Δ. An element of the sequence $\Delta^{L}$ is assigned to the region (x,y) of the lighting grid by the function $\xi^{xy}$:

(29) $$\xi^{xy}\left(ROG\left(x,y\right),\Delta \right)=\frac{\Delta (x\;\mathrm{mod}\;n)+\Delta (y\;\mathrm{mod}\;n)}{2}$$

Finally, the function ‘*Interlaced XY*’ is defined as meeting a spatial gradient in two axes of the grid Equation (22) and an amplitude gradient formed by the *Interlaced* configuration Equation (24). This function uses the ordered sequence $\Delta^{E}$ Equation (28) of the set Δ. An element of the sequence $\Delta^{L}$ is assigned to the region (x,y) of the grid by the function $\xi^{xy}$ Equation (29).

Also, a reference lighting configuration is defined. It permits the comparison of the improvement produced by enhancing the target parameters to measure. In this case, all the regions of the grid have the same characteristics. The set Δ is built with one element (monochromatic or polychromatic lights).

(30) Δ=δ,ξ(ROG(x,y))=δ

**Figure 12:**
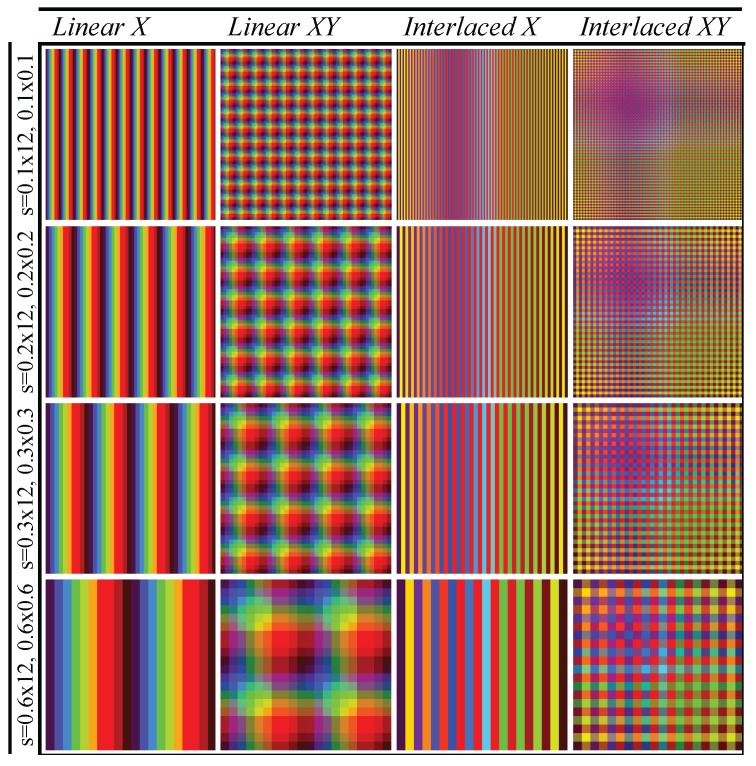
Samples of lighting patterns generated by the transformation $\upsilon_{\Phi }$ used in the experimentation.

The parameters used in the transformations $\Upsilon_{\Phi }$ are summarized below (see Table 3). The lighting covers the whole surface (12 mm × 12 mm). The areas of ROLs generated by *s* are 0.1 mm × 12 mm, 0.2 mm × 12 mm, 0.3 mm × 12 mm and 0.6 mm × 12 mm for the functions ‘*Linear X*’ and *Interlaced X*. The ‘*Linear XY*’ and ‘*Interlaced XY*’ functions from ROL have areas of 0.1 mm × 0.1 mm, 0.2 mm × 0.2 mm, 0.3 mm × 0.3 mm and 0.6 mm × 0.6 mm. According to lighting characteristics Δ, ‘*Linear X*’ and ‘*Linear XY*’ use the sequence $\Delta^{L}$ Equation (25) with 10 elements for all cases whereas *Interlaced X* and ’*Interlaced XY*’ use the sequence $\Delta^{E}$ Equation (28) with 120, 60, 40 and 20 wavelengths uniformly distributed from the visible electromagnetic spectrum (380 nm to 780 nm). Finally, the function $\xi^{xy}$ is used for ‘*Linear XY*’ and ‘*Interlaced XY*’. It establishes (120 × 120), (60 × 60), (40 × 40) and (20 × 20) ROLs on the plane. ‘*Linear X*’ and *Interlaced X* are made up using the function $\xi^{x}$ establishing 120, 60, 40 and 20 ROLs according to the areas of the regions considered. Figure 12 shows the different lighting patterns used in the experiments.

### 5.2. Performance Results

In order to obtain the performance results, the components $\rho_{i}$ Equation (2) considered are scale $\rho_{E}$, angle $\rho_{\theta }$ and intensity lighting $\rho_{I}$. They are the most influential scene variables in the image formation and in the characteristics of the visual inspection systems.

The system is tuned to a set of scale values $\rho_{E}$: 1 pixel/mm, 2 pixels/mm, 5 pixels/mm, 10 pixels/mm and 15 pixels/mm. The set of the component angle $\rho_{\theta }$, as the angle formed by the surface normal and the camera normal, is formed by the sequence from 0${}^{\circ }$ to 90${}^{\circ }$ with an increase of 10${}^{\circ }$ (10 different angles in total). Finally, the intensity lighting $\rho_{I}$ is the basic parameter measured by the camera and is related to the solution proposed for increasing the perception capacity of the system. A large set of camera, environment and object variables take part in the formation of this magnitude. The goal of the tests is to study the intensity lighting according to environment values related to the proposed transformations $\Upsilon_{\Phi }$ and not to influence any other variables. However, the angle formed by the surface normal and the lighting plane normal is very important in the design of inspection systems. It affects the intensity lighting $\rho_{I}$. Therefore, it is considered as this parameter. The values contemplated in the tests are from 0${}^{\circ }$ to 90${}^{\circ }$ with differences of 10${}^{\circ }$ (10 different angles in total).

In order to measure the effectiveness of the transformation $\Upsilon_{\Phi }$ to inspect, the performance is calculated as the number of different pixels between the image of an object without defects and the image of the same object with defects. Specifically, this difference divided by the estimated number of pixels containing the defects, for a specific resolution, measures the success rate. The calculation of this rate is performed in all the possible combinations of the variables $\rho_{i}$ contemplated previously. A total of 68,000 images have been synthesized. That is, 17,000 images from objects without defects $M_{P}$ and 51,000 (17,000 × 3 types of defects) images from inspection objects $M_{I}$ with 3 types of defects (2 in topography and 1 in colour). The 17,000 images are the images of 17 lighting configurations (4 $\Upsilon_{\Phi }$ functions of environment condition using 4 different lighting areas defined by *s* and a reference lighting configuration Equation (30)) conditioning the measure of 2 objects (dielectric and metallic) for 500 values of vector ρ (5 $\rho_{E}$, 10 $\rho_{\theta }$ and 10 $\rho_{I}$).

The experimental results of the average success rates for scale $\rho_{E}$, angle $\rho_{\theta }$, lighting $\rho_{I}$ and the influence of the size of lighting *s* regions are detailed in the next sections.

#### 5.2.1. Scale of Perception

The study of the perception scale makes discerning the influence of the size of the defects in the image possible.

**Figure 13:**
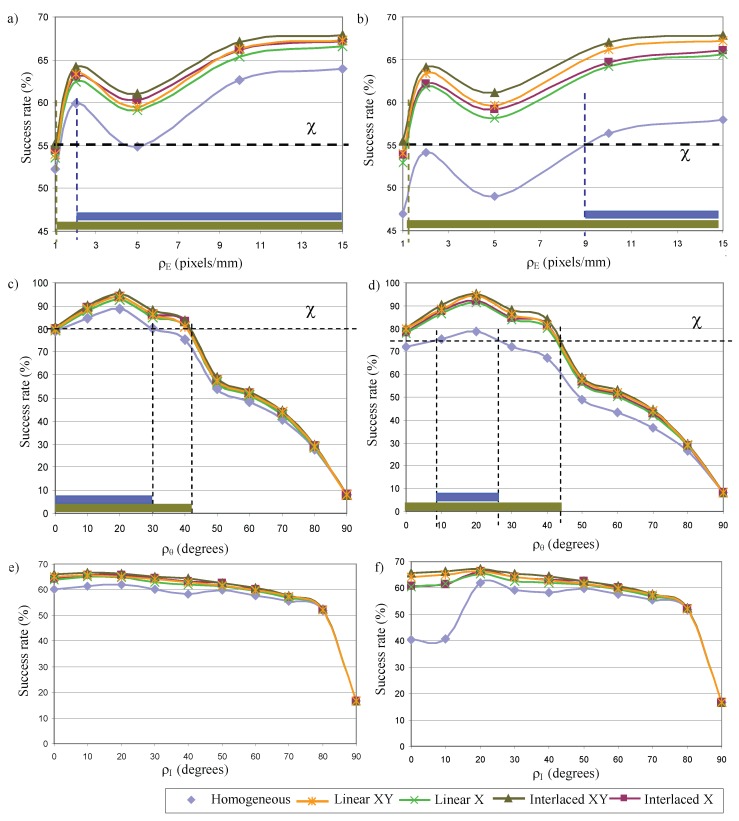
Success rates for the dielectric (**a**,**c**,**e**) and metallic (**b**,**d**,**f**) material according to scale (**a**,**b**), angle (**c**,**d**) and intensity lighting (**e**,**f**).

Figure 13a shows the success rates according to the dielectric object. The function ’*Interlaced XY*’ offers the best success rates whereas the ’*Linear X*’ offers the minimum of the transformations $\Upsilon_{\Phi }$. The average difference between the best function and the reference is 4.33%. The data from functions (’*Linear XY*’ and ’*Interlaced X*’) that uses only one of the maximum proposed gradients, scale or amplitude, is very similar. The average difference is only 0.16%.

The differences in success rates according to metallic object are more noticeable (see Figure 13a). The function ’*Interlaced XY*’ shows similar success rates to the case of dielectric material. The values are practically independent of the type of material. However, the success rates of the reference lighting significantly decrease to values between 46% and 57.9%. This is an average difference of 5.85% lower than the success rate of the dielectric case. Therefore, the improvement of the capacity of perception of the system is better using the alternative *Measurement Enhancement*. It differs more than 10% using the best function (*Interlaced XY*). Also, the results show an increase in sensitivity using spatial distributions organized in two directions ($\xi^{xy}$).

It is interesting to consider the shape of the curve in Figure 13a. The graph represents the success rate of the system as a function of the scale. This is pixels per millimetre and not pixels per defect. The defects used in the tests have a maximum size of 0.6 mm for one of their three dimensions. Therefore, the scale values $\rho_{E}$ correspond to 0.6, 1.2, 3, 6 and 9 pixels per dimension of the defect. For the minimum scale (1 pixel/mm), a crack or crater defect is projected into 0.28 pixels${}^{2}$ and a chromatic defect is projected into 0.36 pixels${}^{2}$. This corresponds to an area of 7.065 and 9 pixels${}^{2}$ respectively in the image of a defective plane (25 defects, see Figure 9). Then, the differences of 1 pixel in the image suppose that success rates vary 11.11% or 14.15%. The ratio of success rate to pixel is very large. Conditions for the next scale (2 pixels/mm) are similar. In this case, differences of 1 pixel in the image means the detection or not of a defect. The topographic defects are projected into an area of 1.13 pixels${}^{2}$ and the chromatic ones into an area of 1.44 pixels${}^{2}$. Differences of 1 pixel for the rest of the scale values mean a lesser impact on the detection.

In short, the average success rates show higher scale $\rho_{E}$, more capacity of perception of the defects both for dielectric and metallic materials, avoiding short scales (1 pixel/mm and 2 pixels/mm) due to the characteristics mentioned in the previous paragraph.

Comparing the functions $\Upsilon_{\Phi }$ and the reference function, according to the capacity of perception, the data shows that a greater adjustment of the scale is required using the latter lighting. For example, if the minimum threshold χ for determining the defect in the dielectric material (this parameter will depend on the type of application) is 55%. Using the reference lighting, approximately more than 2 pixels/mm are necessary to perceive the defects whereas with ’*Interlaced XY*’ lighting only 1 pixel/mm is needed. In the case of metals, greater adjustment of the system is required. For the same threshold χ, more than 9 pixels/mm would be necessary using reference lighting and only 2 pixel/mm for each of the functions $\Upsilon_{\Phi }$ proposed. The best lighting, ’*Interlaced XY*’, makes a system tune to only 1 pixel/mm to detect the possible defects.

#### 5.2.2. Angle of Perception

The success rates of the different transformations $\Upsilon_{\Phi }$ according to the perception angle $\rho_{\theta }$ are shown in Figure 13c,d, for the dielectric and metallic objects respectively.

The study of the average of the success rates shows that the best conditions to perceive according to $\rho_{\theta }$ are in the interval [0${}^{\circ }$, 40${}^{\circ }$] for all lighting configurations. The function ’*Linear X*’ offers the worst results of the transformations $\Upsilon_{\Phi }$ even though their differences are minimum, an average of 2.85%. The functions ’*Linear XY*’ and ’*Interlaced X*’ present a similar behaviour. The average difference between them is 0.77% and it reaches a maximum of 1.12% for an angle of 20${}^{\circ }$.

The best results are provided by the function ’*Interlaced XY*’. The improvement in the capacity of perception reaches the maximum differences, an average difference of 11.4%, in the interval [10${}^{\circ }$, 40${}^{\circ }$] compared to using the reference lighting. According to the material type in this interval, the differences are 6.84% and 16.05% for the dielectric and metallic case respectively. This is due to the fact that the success rates significantly decrease from inspecting dielectrics materials to metallic one, an average of 8.99% in the interval. Also, the differences in the success rates between the transformations $\Upsilon_{\Phi }$ that light the metallic objects are more noticeable.

An angle of 20${}^{\circ }$ formed by the surface normal and the camera normal sets the maximum success rate. Using the function ’*Interlaced XY*’ for this angle, similar results are obtained according to the type of material: 95.05% for dielectric materials and 95.19% for metallic one. Furthermore, for the metallic object in this angle, the difference between the ’*Interlaced XY*’ and the lighting reference is 16.53%.

The success rate and the differences between the transformations and reference lighting decrease from 40${}^{\circ }$ to reach 8.2% at 90${}^{\circ }$. In the latter case, it is only possible to detect convex defects.

Comparing the functions $\Upsilon_{\Phi }$ and the reference function, according to the capacity of perception, the data shows that the increase in the number of the magnitudes of a scene which the system is able to perceive is more significant than in the study of the scale. For example, if the minimum threshold χ for determining the defect in the dielectric material is 80% using the reference lighting, the angle $\rho_{\theta }$ must be less than 30${}^{\circ }$ to perceive the defects. In this case, using the ’*Interlaced XY*’ lighting, the angle can increase by up to approximately 43${}^{\circ }$. Hence, the increase is more than 10${}^{\circ }$. Assuming a threshold of 75%, the angle formed by the surface normal and the camera normal can be established in the interval [10${}^{\circ }$–25${}^{\circ }$] for determining the defect in the metallic material using the reference lighting. Using any transformation $\Upsilon_{\Phi }$, the angle tuning can be established in the interval [0${}^{\circ }$–45${}^{\circ }$]. Then, the increase is about 30${}^{\circ }$.

#### 5.2.3. Intensity Lighting

Regarding the intensity lighting $\rho_{I}$ as the angle formed by surface normal and lighting plane normal, the success rates of the different lighting configurations are shown in the Figure 13e,f, for the dielectric and metallic objects respectively. The function ’*Interlaced XY*’ provides the maximum success rate and the greatest increase in the capacity of perception of the system.

The study of the inspection capacity considering the material type shows the limited sensitivity for perceiving metallic objects using angles $\rho_{I}$ close to 0${}^{\circ }$ and the reference lighting. The characteristics of the lighting sources and of the metallic reflection cause sensor saturation in the angle interval [0${}^{\circ }$, 10${}^{\circ }$]. The decreasing of the success rate, compared to the dielectric material, is considerably marked with values close to 20% (concretely 19.72% for 0${}^{\circ }$ and 20.77% for 10${}^{\circ }$). Also, transformations using minimum spatial differences show differences of more than 3% between metallic and dielectric in that interval: ’*Interlaced X*’ 3.30% (0${}^{\circ }$) and 4.36% (10${}^{\circ }$), ’*Linear X*’ 3.47% (0${}^{\circ }$) and 3.30% (10${}^{\circ }$). However, if the transformations with spatial distributions in two dimensions are used, sensor saturation is not important. The differences are 0.33% (0${}^{\circ }$) and 0.45% (10${}^{\circ }$) for the function ’*Interlaced XY*’ and 0.73% (0${}^{\circ }$) and 0.37% (0${}^{\circ }$) for ’*Linear XY*’. The success rate is independent of the material and the differences are not significant for the rest of the lighting angles.

The success rate shows a low gradient for the intensity lighting $\rho_{I}$ according to lighting angle. The magnitude has an almost constant behaviour in the interval [0${}^{\circ }$, 60${}^{\circ }$]. The standard deviation is lower than 2 for the transformations $\Upsilon_{\Phi }$ whereas the value is 4.21 for the reference lighting for all angles due to the behaviour in the initial values of the metallic plane (the standard deviation for the dielectric plane is 1.44).

#### 5.2.4. Size of Lighting Regions

**Figure 14:**
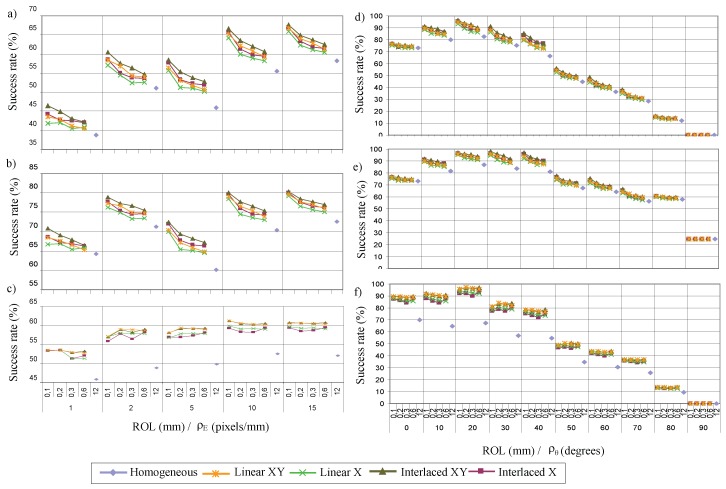
Success rates for inspecting a crater (**a**,**d**), crack (**b**,**e**) and chromatic defect (**c**,**f**) according to size of lighting regions and scale (**a**–**c**) and angle of perception (**d**–**f**).

The influence of the size of the lighting regions ROL defined by *s* on the capacity of perception according to the scale is indicated on the left of Figure 14 and according to the angle on the right of the Figure 14. The size of ROL is represented by the minimum dimension of the region.

Increasing the size of ROL decreases the rate of the regions inside the defect. This fact decreases the success rates of inspecting topographic defects using the transformations $\Upsilon_{\Phi }$ for all perception scales and angles. The average differences vary from 4% to 5% in the scale case. According to angle, the average differences are about 7% for the crack defect (Figure 14e) in the interval of the maximum sensitivity [10${}^{\circ }$ , 40${}^{\circ }$]. The behaviour for the crater defect (Figure 14d) is analogous; it establishes an average difference of sensitivity in that interval of 5%. The differences decrease as the perception angle increases until they are 0 for 90${}^{\circ }$.

The capacity to modulate irradiance in the defect is inversely proportional to its size. In the extreme case where the size of the bands is greater than the size of the defects, the irradiance function will not be able to modulate the reflectance of the object, so it would behave in the same way as a the reference lighting.

The analysis of the chromatic defect shows that the size of lighting regions is independent of the success rate (see Figure 14c,f). In this case, it is practically constant. Therefore, a considerable increase in the perception capacity of the system is obtained.

## 6. Case Study: Automobile Logo

Simulation, as a previous step of experimentation in real manufacturing environments, allows a preliminary study that can be used to discern the best acquisition path planning or the equipment characteristics to perform the inspection of the specular surfaces. Finally, in this section an example of the application of the experimental results of the scale $\rho_{E}$ and angle $\rho_{\theta }$ of perception on specular surface acquisition for inspecting a 100 mm diameter Mercedes Benz metallic logo is shown. The area to be inspected is 4676.15 mm${}^{2}$. Given that the minimum threshold χ depends on the application, in this case a value of 80% is assumed.

**Figure 15:**
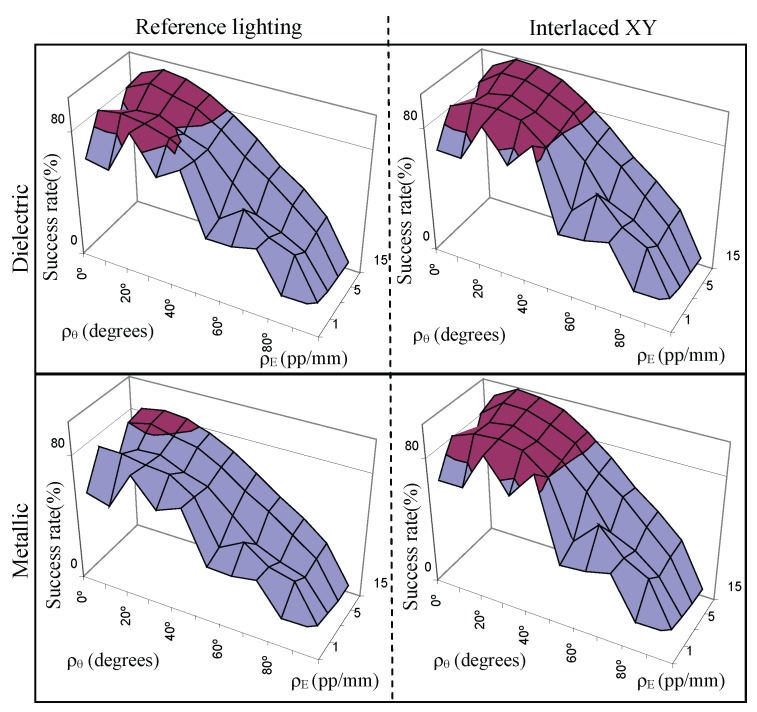
Success rates for the dielectric and metallic surfaces according to lighting of reference and the transformation $\Upsilon_{\Phi }$ with best results (’*Interlaced XY*’).

The inspection conditions are restricted to only 5 values of the scene magnitudes $\rho_{i}$ for a metallic object with an unstructured lighting (reference lighting). As can be seen in Figure 15 considering scale and angle ($\rho_{\theta }$, $\rho_{E}$), the scene magnitudes are the following: (0${}^{\circ }$, 2 pixels/mm), (10${}^{\circ }$, 10 pixels/mm) and those with the considered highest scale ([0${}^{\circ }$, 10${}^{\circ }$, 20${}^{\circ }$], 15 pixels/mm). The best lighting configuration, $\Upsilon_{\Phi }$ ’*Interlaced XY*’, permits increase the tuning of the scene magnitudes system up to 20 points (see Figure 15): ([20${}^{\circ }$, 40${}^{\circ }$], 1 pixel/mm), ([0${}^{\circ }$, 10${}^{\circ }$, 20${}^{\circ }$, 30${}^{\circ }$], 2 pixels/mm), ([10${}^{\circ }$, 20${}^{\circ }$, 30${}^{\circ }$, 40^o^], 5 pixels/mm), ([0${}^{\circ }$, 10${}^{\circ }$, 20${}^{\circ }$, 30${}^{\circ }$, 40${}^{\circ }$], [10, 15] pixels/mm).

The choice of the conditions (scale, angles, etc.) to capture the whole object for inspection is a complex problem. It is necessary to take into account the particularities of each solution. In this case as example, the scale $\rho_{E}$ is established at 15 pixels/mm. Therefore, according to the previous scene magnitudes, the perception angle can deviate by up to 20${}^{\circ }$ using the reference lighting and by up to 40${}^{\circ }$ using the function ’*Interlaced XY*’. In other words, the angle formed by the vector normal to the camera and the normal vector of the surface to be inspected must be from 0${}^{\circ }$ up to 20${}^{\circ }$ using the reference ligthing and from 0${}^{\circ }$ up to 40${}^{\circ }$ in case of ’*Interlaced XY*’ lighting is used.

**Figure 16:**
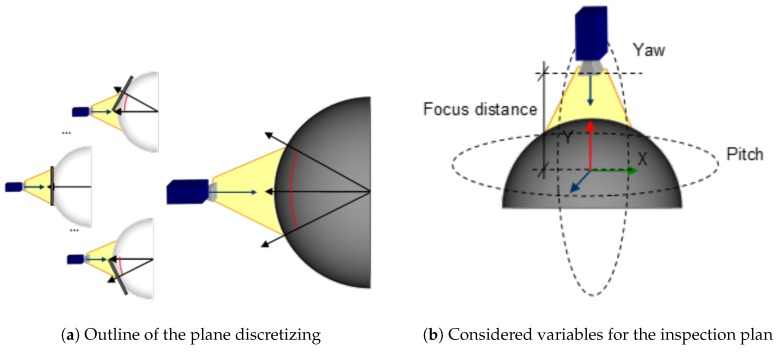
Essential considerations for transferring the conclusions of experimental values to a specific inspection of an object.

In consequence, in order to apply the results, any point on the surface can be viewed as a point on a plane whose normal vector is the normal of the surface at that point. For example, Figure 16a shows an outline of this assumption. In this way, the surface can be analysed as a set of planes (a plane per point on the surface). Then, the experimental results (calculated for planes) can be extrapolated to calculate the perception scale and angle of surfaces of different curvature for any point on the surface. The use of planes for any point on the surface could be computationally expensive. Hence, as the function $\mu_{S}$ establishes the points on the surface in a coordinate system that is local to the object, in practice the function $\mu_{S}$ is defined as a triangle mesh: a collection of triangles that defines the surface shape of a polyhedral object in 3D computer graphics. The use of a triangle mesh allows the system to discretize the surface geometry as a reduced collection of planes. The number of planar faces will be determined by the geometry of the surface and the resolution used in the experiments (in this case more than 4000 polygons, although less triangles are enough, high details are not needed due to angle perception is discretized each 10${}^{\circ }$).

Since the scene magnitudes are a function of the characteristics of the object, the environment and the camera, it is required to make decisions about the appropriate magnitudes to infer to provide them. In other words, it is necessary to decide which magnitudes of the environment or the camera have to be modified to establish the adequate values of scale and angles calculated in the experiments. The scale will be determined by the number of pixels available to the sensor by setting the camera position at a focus distance and at a constant focal length. The conditions of the angle of perception will be established by the movement on the *X* and *Y* axis of the origin of coordinates located in the center of the of the object: the Yaw and Pitch movements of the camera (see Figure 16b). Due to select the variables is a complex problem, an approximation to the optimum solution is proposed in order to determine the appropriate angles between camera and object surface. This increases the captured area of the logo and reduces the images to be captured. For this purpose, a search tree was designed using a branch and bound algorithm, in which the solutions space of each node is reduced to a maximum of five children and a maximum temporal processing level is established.

**Figure 17:**
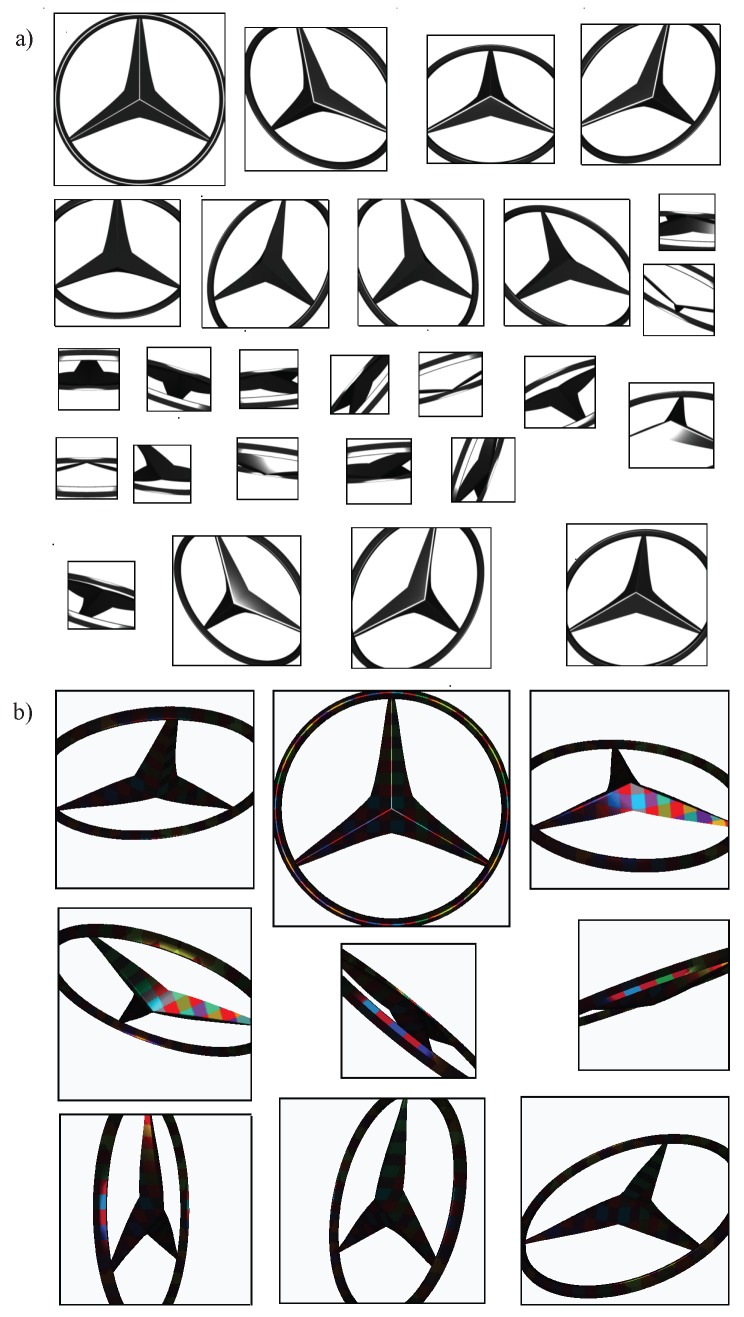
Images of a metallic logo to be inspected using the reference lighting (**a**) and the function ’*Interlaced XY*’ (**b**).

The logo inspection requires 26 captures (see Figure 17a and Table 4) using the lighting reference and a camera of 1452 × 1452 pixels to acquire a surface area of 4619.96 mm${}^{2}$ (98.79% of the logo). Some captures can be made using a lower resolution of up to 510 × 510 pixels. According to the function ’*Interlaced XY*’, only 9 images are necessary (see Figure 17b and Table 5). The camera resolution varies between 826 × 826 and 1455 × 1455 in order to cover a total surface area of 4675.05 mm${}^{2}$ (99.9%). The first capture obtains 45% of the total inspection of the logo whereas only 8.17% is obtained using the reference lighting. This fact shows how the lighting system allow to perceive in more scene magnitudes. Specifically, more points on the surface accomplish the angle of perception $\rho_{\theta }$.

## 7. Conclusions

A novel active imaging model able to increase the perception capacity of the visual system is presented. The model provides solutions to vision problems in which it is difficult to perceive. It describes the parameters involved in a visual sensorization system in three main aspects: target object to perceive, environmental conditions, and sensor parameters. Each of them is individually parameterized as object size, color, etc.; environment parameters as light, objects-sensor orientations,...; and sensor parametrization such as focal length, monochrome/RGB/3D, etc. Moreover, the model describes the perception capabilities and the limits, and present solutions to these limits. In particular, the model is instantiated for the specular object inspection, as an example of a challenging situation for visual sensor perception. The specular surfaces problem means the device must operate in the intervals of low perception, related to reflections and shine. Traditionally, automated visual inspection requires a thorough analysis of the problem where solutions include everything from the acquisition equipment to the algorithm to recognize the possible defects. As a consequence, these vision systems are oriented to concrete applications and cannot be generalized. The model presented here deals with this lack by providing a general representation of vision systems and solutions for the perception limitations.

The solution proposal of the problem related to specular surfaces provides a normalization of the image in which different objects perceived as the same can be distinguished. First, *Measurement Enhancement* increasing the differences of the input magnitudes is performed. In this paper, the enhancement of measurements is carried out using environment variables, concretely controlling the lighting conditions. This enhancement spatially structures the lighting in order to set up regions on the surface of the object radiated using different spectral powers forming a grid. Finally, the system is tuned in order for the magnitudes of the scene (scale, angles, intensity lighting, etc.) to be properly perceived.

According to thorough analysis of the problem characteristics, use of virtual imaging simulations as a preliminary method step for validating hypotheses of the visual inspection systems is proposed. The validation of the conditions (point of view, scale, lighting, etc.) in which the inspection has to be performed, can be carried out in a flexible and low cost manner justifying the use of simulations in an early warning step of the system viability. Hence, the model can be pre-validated before the system is developed.

The use of simulations and knowledge bases and the generalist approach of the transformation provide a general solution that can be systematically applied. The method can be applied to the resolution of different inspection problems adapting the contents of the knowledge bases, avoiding a new design solution for each problem.

A realistic simulator has been designed to carry out the experimentation. This simulator recreates the conditions of the image formation and permits the validation of the inspection systems based on the model. The test shows the use of the transformations $\Upsilon_{\Phi }$ improves the capacity of perception of the system compared to a homogeneous environment (only one wavelength or colour). This is both for dielectric and metallic materials with regard to the tuning of the scale, angle or lighting. The use of transformation considering maximum amplitude gradient and maximum spatial differences (’*Interlaced XY*’) obtains the best results to perceive the defects for all cases. In contrast, the function with minimum amplitude gradient and minimum spatial differences (’*Linear X*’) offers the worst results of the transformations considered to perform the *Measurement Enhancement*. The functions (’*Linear XY*’ and ’*Interlaced X*’) that use only one of the maximum proposed gradients, scale or amplitude, generally present similar behaviour. The function that performs a homogeneous lighting of the object surface, which is used as reference, obtains the minimum success rate in all cases. Hence, the results prove the improvement in the capacity of perception for different conditions of scale, angle and intensity lighting. The proposed method enables the detection of surface defects in a greater number of values of scale, angle of perception and lighting conditions than in normal conditions using uniform lighting. The immediate repercussion is that a smaller number of captures of the scene is needed by the system.

The research should continue by studying the *Measurement Enhancement* by means of the extension of the input magnitudes: using other transformations based on structured lighting (with different patterns and using the time domain) and using other parameters like variables of the capture system to provide great gradients in the image.

The simulation confirms the hypotheses. It would be wise to advance to physical experiments according to the concrete industry in which the system is to be technologically developed. Nowadays, the ’*Interlaced XY*’ function (in this case, the pattern is made up using the ordered sequence $\Delta^{E}$ of grey levels instead of using wavelengths) is being tested for increasing the perception capacity of an inspection system aimed to detect shape defects on the surfaces of ceramic tiles.

## Figures and Tables

**Table 1:** Characterists of the objects to be generated in the experiments.

Characteristic	Metallic	Dielectric
Surface roughness (rms)	0.1	
Refraction index	2.8	1.6
Extinction index	3.2	0
Specularity coefficient	0.75	
Diffuse component (RGB)	(0.6,0.6,0.6)	
Diffuse component defect (RGB)	(0.4,0.4,0.4)	
Object size (mm)	12 × 12	
Defect size (mm)	0.6 diameter or side	

**Table 2:** Lighting configuration using combinations of the spatial and amplitude gradients.

		Spatial Gradient	
		X ($\xi_{x}$)	XY ($\xi_{\mathrm{xy}}$)
Amplitude gradient	Linear ($\xi_{L}$)	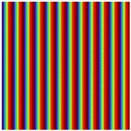	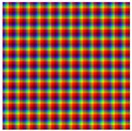
		$\xi_{L,x}$	$\xi_{L,xy}$
	Interlazed ($\xi_{I}$)	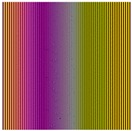	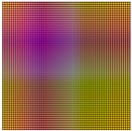
		$\xi_{I,x}$	$\xi_{I,xy}$

**Table 3:** Parameters for the transformations $\Upsilon_{\Phi }$ used in experiments.

Characteristics	Linear X	Linear XY	Interlazed X	Interlazed XY
*s*: ROL areas (mm)	0.1 × 12	0.1 × 0.1	0.1 × 12	0.1 × 0.1
	0.2 × 12	0.2 × 0.2	0.2 × 12	0.2 × 0.2
	0.3 × 12	0.3 × 0.3	0.3 × 12	0.3 × 0.3
	0.6 × 12	0.6 × 0.6	0.6 × 12	0.6 × 0.6
*s*: ROG areas (mm)	0.1 × 0.1			
	0.2 × 0.2			
	0.3 × 0.3			
	0.6 × 0.6			
Δ (# wavelenghts)	10		120	
			60	
			40	
			20	

**Table 4:** Environment and camera characteristics to acquire 26 images needed to inspect the metallic logo using the reference lighting.

Yaw/Pitch	CCD	Image Area	Inspected Area	Accumulated Area	Inspected
(degrees)	(pixels)	(mm${}^{2}$)	(mm${}^{2}$)	(mm${}^{2}$)	(%)
(0, 0)	1452 × 1452	9377.45	381.97	381.97	8.17%
(30, −30)	1218 × 1218	6588.08	769.21	1151.19	24.62%
(0, 40)	1186 × 1186	5982.37	761.16	1912.35	40.90%
(−30, −30)	1215 × 1215	6503.9	758.21	2670.56	57.11%
(40, 20)	1167 × 1167	5691.68	222.63	2893.19	61.87%
(−40, 20)	1166 × 1166	5680.73	220.89	3114.07	66.60%
(0, −80)	550 × 550	707.26	143.46	3257.53	69.66%
(−60, 70)	570 × 570	1038.1	142.98	3400.51	72.72%
(30, 80)	510 × 510	630.42	142.09	3542.6	75.76%
(80, 40)	519 × 519	1127.5	141.35	3683.95	78.78%
(−60, −70)	570 × 570	1015.71	140.94	3824.89	81.80%
(−20, 80)	493 × 493	620.97	138	3962.89	84.75%
(0, −40)	1137 × 1137	4144.17	137.76	4100.66	87.69%
(50, −80)	543 × 543	615.36	131.11	4231.77	90.50%
(−30, −80)	560 × 560	705.12	88.05	4319.82	92.38%
(−80, −20)	576 × 576	1075.71	67.33	4387.15	93.82%
(50, 60)	635 × 635	1744.96	47.76	4434.91	94.84%
(0, 80)	550 × 550	695.4	45.85	4480.76	95.82%
(20, −70)	526 × 526	1213.01	32.72	4513.49	96.52%
(10, 30)	1280 × 1280	6672.88	31	4544.48	97.18%
(60, −60)	630 × 630	1538.01	29.37	4573.85	97.81%
(−30, 40)	1133 × 1133	5565.17	25.12	4598.97	98.35%
(−50, 70)	586 × 586	1177.92	11.57	4610.55	98.60%
(40, −30)	1164 × 1164	5933.79	4.83	4615.37	98.70%
(−40, −20)	1192 × 1192	6175.95	4.41	4619.79	98.80%
(10, 60)	749 × 749	1970.75	0.17	4619.96	98.80%

**Table 5:** Environment and camera characteristics to acquire nine images needed to inspect the metallic logo using ’*Interlaced XY*’ lighting.

Yaw/Pitch	CCD	Image Area	Inspected Area	Accumulated Area	Inspected
(degrees)	(pixels)	(mm${}^{2}$)	(mm${}^{2}$)	(mm${}^{2}$)	(%)
(0, 0)	1455 × 1455	9410.15	2079.07	2079.07	44.46%
(−10, −60)	1219 × 1219	4252.76	581.96	2661.04	56.91%
(−10, 60)	1221 × 1221	4234.16	580.29	3241.33	69.32%
(60, 10)	1263 × 1263	4574.35	560.57	3801.9	81.30%
(−70, 0)	1174 × 1174	2899.19	480.71	4282.61	91.58%
(40, −60)	1187 × 1187	4118.58	197.45	4480.06	95.81%
(30, 50)	1279 × 1279	5563.56	121.89	4601.95	98.41%
(−80, 50)	826 × 826	2711.22	36.55	4638.5	99.20%
(−80, −70)	923 × 923	1695.65	36.55	4675.05	99.98%
